# Multicentric Carpotarsal Osteolysis Syndrome Associated Nephropathy: Novel Variants of *MAFB* Gene and Literature Review

**DOI:** 10.3390/jcm11154423

**Published:** 2022-07-29

**Authors:** Stefania Drovandi, Francesca Lugani, Olivia Boyer, Edoardo La Porta, Paolo Giordano, Aurélie Hummel, Bertrand Knebelmann, Joséphine Cornet, Genevieve Baujat, Beata S. Lipska-Ziętkiewicz, Gian Marco Ghiggeri, Gianluca Caridi, Andrea Angeletti

**Affiliations:** 1 Division of Nephrology, Dialysis, and Transplantation, IRCCS Istituto Giannina Gaslini, 16147 Genoa, Italy; stefania.drovandi@gmail.com (S.D.); edoardolaporta@gaslini.org (E.L.P.); plo.grd@gmail.com (P.G.); gmarcoghiggeri@gaslini.org (G.M.G.); 2 Laboratory of Molecular Nephrology, IRCCS Istituto Giannina Gaslini, 16147 Genoa, Italy; francescalugani@gaslini.org (F.L.); gianlucacaridi@gaslini.org (G.C.); 3 PHP, Service de Néphrologie Pédiatrique, Institut Imagine, Centre de Référence MARHEA, Hôpital Universitaire Necker-Enfants Malades, Université Paris Cité, 75015 Paris, France; olivia.boyer@aphp.fr (O.B.); aurelie.hummel@aphp.fr (A.H.); 4 Nephrology Department, Reference Center for Inherited Kidney Diseases (MARHEA), APHP, Necker Hospital, Paris University, 75015 Paris, France; bertrand.knebelmann@aphp.fr (B.K.); josephine.cornet@aphp.fr (J.C.); 5 Reference Centre for Constitutional Bone Diseases, Laboratory of Osteochondrodysplasia, INSERM UMR 1163, Imagine Institute, Université de Paris, 75015 Paris, France; genevieve.baujat@nck.aphp.fr; 6 Rare Diseases Centre, Medical University of Gdansk, 80-210 Gdansk, Poland; beata.lipska-zietkiewicz@gumed.edu.pl; 7 Department of Biology and Medical Genetics, Clinical Genetics Unit, Medical University of Gdansk, 80-210 Gdansk, Poland

**Keywords:** multicentric carpotarsal syndrome, monogenic kidney disease, hereditary podocytopathy, glomerulonephritis, nephrotic syndrome, renal failure

## Abstract

Multicentric carpo-tarsal osteolysis (MCTO) is a rare osteolysis syndrome mainly involving carpal and tarsal bones usually presenting in early childhood. MCTO has autosomal dominant inheritance with heterozygous mutation in the *MAFB* gene. The skeletal disorder is often associated with chronic kidney disease. Data on clinical characterization and best treatment option of MCTO-associated nephropathy are scarce and mostly limited to case reports. With the aim to better define the phenotype and long-term outcomes of MCTO-associated nephropathy, we launched an online survey through the Workgroup for hereditary glomerulopathies of the European Rare Kidney Disease Network (ERKNet). Overall, we collected clinical and genetic data of 54 MCTO patients, of which 42 previously described and 12 new patients. We observed a high rate of kidney involvement (70%), early age of kidney disease onset, nephrotic-range proteinuria, and a kidney survival around of 40% at long-term follow-up. Our finding confirmed the heterogeneity of clinical manifestations and widen the spectrum of phenotypes resulting from MCTO-associated nephropathy. Furthermore, we report the first case of complete remission after treatment with cyclosporine A. We demonstrated that multidisciplinary care is essential for MCTO patients and early referral to nephrologists is therefore warranted to facilitate prompt treatment.

## 1. Introduction

Multicentric carpo-tarsal osteolysis syndrome (MCTO;OMIM#166300) is an ultrarare autosomal dominant skeletal disorder caused by mutations in the *MAFB* gene (v-maf musculoaponeurotic fibrosarcoma oncogene ortholog B), with high frequency of sporadic cases [1]. MCTO is characterized by progressive osteolysis mainly involving the carpal and tarsal bones: after a variable time (months to years) of normal bone development, patients may present painful and swelling joints, deformities, and progressive articular restriction. MCTO is often misdiagnosed as juvenile idiopathic arthritis (JIA) due to similar clinical manifestations. Subjects affected by MCTO often present kidney involvement, nearly 55% of reported cases, that usually presents with proteinuria and may progress to kidney failure (KF). Furthermore, mental retardation and minor facial deformities have been reported to be associated with MCTO disease.

At now, the rarity of the disease had largely limited a comprehensive characterization of renal involvement; furthermore, a consensus on possible specific treatments is missing. Moreover, mostly due to the primary onset of non-nephrological manifestations, diagnosis of kidney involvement may be delayed with negative consequences in terms of worsening of kidney disease. With the aim to elucidate the phenotype and outcomes of MCTO-associated nephropathy, we here present two-centers retrospective analysis and a comprehensive revision of the literature, for an amount of 54 patients with genetic diagnosis of MCTO. We provide a comprehensive clinical description of cases presentation and clinical outcomes.

## 2. Materials and Methods

### 2.1. Data Collection

**Figure 1:**
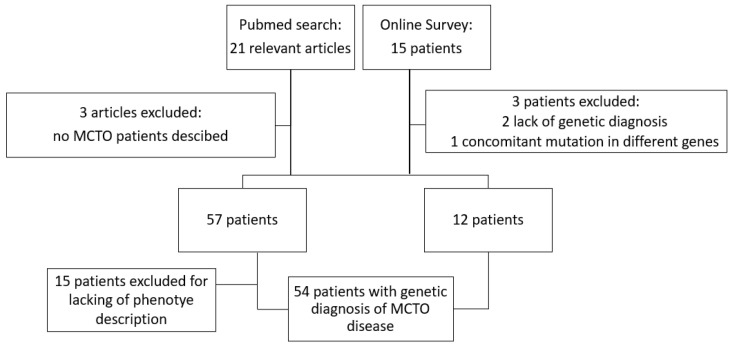
Schematic representation of case selection process.

We compiled the study cohort from two different sources as reported in Figure 1. We performed a systematic literature search on the PubMed database, screening all publications from the inception of the database from 2012 (i.e., year of the identification of *MAFB* as the underlying disease-causing gene for MCTO disease) until May 2022 for the terms “MAFB”, “multicentric carpo tarsal osteolysis”, “MCTO” in all combinations. The references cited in the identified publications were also checked to identify additional papers. Overall, 21 articles of interest were selected on the basis of title and abstract. After removing three publications with no cases reported, 57 cases from 18 articles were identified and a comprehensive phenotype description was available for 42 patients. 

We also proposed an online survey in the context of the Workgroup for hereditary glomerulopathies of the European Rare Kidney Disease Network (ERKNet). Invitations to the online survey were sent to all members of ERKNet. The datasets were completely anonymized: names, initials, date of birth or hospital-specific patient identifiers were not collected (See Supplementary Material S1). Informed consent for data collection and usage for research purpose was provided by the participants’ legal guardian/next of kin at local Centers. A total of 15 patients were reported, from three different pediatric hospitals; we excluded the two patients provided by one of the three pediatric hospitals because of lack of genetic diagnosis (Sanger or Next-Generation Sequencing (NGS)-based diagnosis of autosomal dominant pathogenetic variant in *MAFB* gene) and one patient because of reporting pathogenetic variant in a different gene (*MYH3* gene) involved in bone-kidney disorder.

### 2.2. Statistical Analysis 

Continuous variables are expressed as median and interquartile range (IQR). Discrete variables are expressed as percentages. Survival rates were calculated using Kaplan Meier lifetable analysis, with log-rank testing for analysis of significant differences. Statistical significance was set at *p* < 0.05. Statistical analysis was performed using GraphPad Prism 9.0 software system (GraphPad Software, Inc., La Jolla, CA, USA).

## 3. Results

We collected a cohort of 54 patients affected by MCTO disease, 42 already reported in the literature and 12 patients not previously described on care by two European Pediatric Centers for rare diseases: the Istituto Giannina Gaslini (Genoa, Italy) and the Necker-Enfants Malades Children’s Hospital (Paris, France). A summary of the phenotype characteristics and clinical outcomes are presented in Table 1. 

### 3.1. Genotype

A total of 18 (3 novel) disease-causing variants in MAFB gene were identified and were classified according to the American College of Medical Genetics and Genomics (ACGM) classification (see Supplementary Material Table S1 and Figure S1). All subjects carried missense variants in heterozygous state and all variants lie within a short region of amino-terminal transcriptional activation domain (amino acids 54–71) [1], one subject presented somatic mosaicism [2]. Genetic testing of parents was available in 22 cases: 60% (13/22) resulted sporadic cases (i.e., de novo mutations), 40% (9/22) resulted inherited mutations. Among the parents carrying the mutation whose clinical data were available (8/9), only 25% (2/8) had already received the correct diagnosis of MCTO, 50% (4/8) had previously received a misdiagnosis of arthritis, and 25% (2/8) were asymptomatic carriers, one carried somatic mosaicism.

### 3.2. Bone Disease

Bone manifestations are present in 53/54 of subjects (98.1%), except a single case characterized by somatic mosaicism. The median age of clinical manifestation was 2 years old (IQR 1–4), while the age at genetic diagnosis was 9.7 years (IQR 5.1–15.7) years, resulting in an average diagnostic delay of more than 5 years. The 30% of patients were misdiagnosed as rheumatological disorder and 15% of them received potentially nephrotoxic treatment with non-steroidal anti-inflammatory drugs (NSAIDs). Patients showed a wide spectrum of bone phenotypes, all patients presented upper limbs involvement and around 70% showed lower limbs involvement. The joints/sites affected by the disease were in order of frequency: carpal bones, tarsal bones, elbows, fingers/toes, and knees. One-third of patients showed scoliosis and three patients had cervical deformity or Arnold-Chiari syndrome. 

### 3.3. Kidney Disease

A relevant percentage of patients presented kidney involvement, accounting for the 70% of cases (38/54). Kidney disease was never reported as primitive clinical manifestation and it presented after a median of 5 (IQR 1–11.3) years after bone disease onset. Sixteen patients have not reported occurrence of nephropathy during the follow-up. The median age at last observation was 12 (IQR 6.5–15.2) years.

The age at kidney disease manifestation differed markedly among patients. The median age was 7 (IQR 4–13.7) years. The earliest age of proteinuria onset was 8 months, while the older was 29 years old. Majority of subjects presented with proteinuria, mostly (67%) in sub-nephrotic range. One patient presented with chronic kidney disease stage 2 (eGFR 71 mL/min/1.73 m^2^) and 20% of subject had kidney failure at onset. Microhematuria was reported in a single case.

A kidney biopsy was performed in 28% (11 of 38 patients with kidney involvement) of cases and focal segmental glomerulosclerosis (FSGS) was the most frequent histological finding (82%). The remaining cases were reported as mesangial proliferation/hypercellularity and tubule-interstitial damage, respectively. Direct immunofluorescence microscopy detected non-specific deposits in glomerular basement membranes and/or mesangium. 

**Figure 2:**
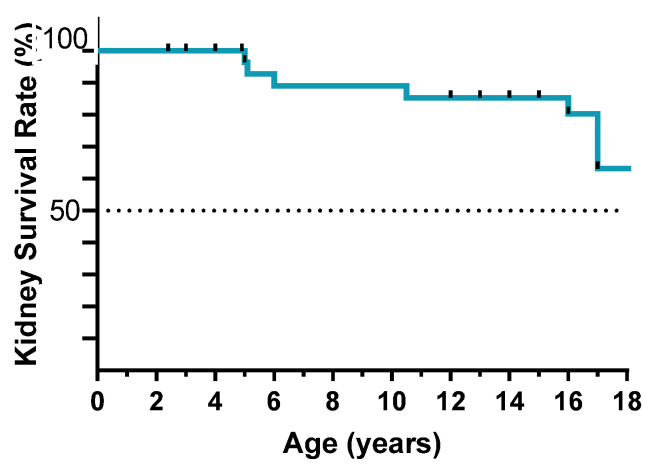
Kidney failure-free survival until adulthood.

The median follow-up from diagnosis of kidney disease was 9 years (IQR 3–13), with a median age at last observation of 15 years (IQR 13–15.2), 63% of patients reach adulthood (18 years of age) with preserved kidney function (Figure 2) and 55% had normal kidney function at the last follow-up. Seventeen patients progressed to kidney failure during the follow-up within the third decade of life. The median age at ESRD was 17 years (IQR 9.3–20). At the last follow-up, 13 patients had received a kidney transplant (one of them received two kidney transplants due to graft lost for chronic transplant glomerulopathy of the first graft), whereas 5 patients were in dialysis (peritoneal dialysis or hemodialysis). As expected, disease recurrence in the allograft was never observed. 

One affected female presented preeclampsia during pregnancies. Two patients presented congenital anomalies of the kidney and urinary tract (CAKUT), characterized by severe bilateral vesicoureteral reflux and/or unilateral hypoplastic kidney. In one case, the urinary malformation was associated with proteinuria and showed mesangial and tubulointerstitial anomalies at the kidney biopsy.

### 3.4. Renal Treatment

**Figure 3:**
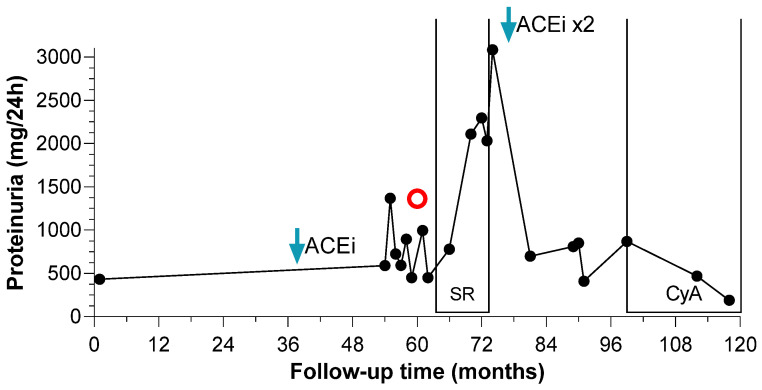
Proteinuria changes over time of CyA-sensitive patient. Proteinuria (mg/day) is reported over time (months) since the diagnosis of MCTO disorder. Red circle corresponds to the kidney biopsy.

Oral steroids were administered in two subjects with proteinuria, with consequent steroid-resistance. The 30% of patients with kidney involvement were treated with renin-angiotensin-aldosterone system inhibitors (RAASi) with partial or complete remission of proteinuria in 66.6% of cases. Oral steroids were administered in two subjects with proteinuria, reporting steroid-resistance. One of them achieved partial remission after maximization of RAASi; he was then treated with cyclosporine (Cya), showing complete remission of proteinuria (Figure 3). Renal function and serum albumin remained always in normal range.

When proteinuria exceeded 500 mg/24 h the patient started ACEi (Enalapril 5 mg daily) (blue arrow). Due to worsening of proteinuria, a kidney biopsy was performed at month 60 (red circle), with the diagnosis of focal segmental glomeruloscelrosis. The patient received 8-months course of oral steroids without benefit. The dose of ACEi was doubled at month 73 (blue arrow) and the proteinuria set around 600–700 mg/24 h. The patient started cyclosporine (150 mg daily) at month 98 (CyA brackets) and over 11 months the proteinuria decreased below 200 mg/24 h (190 mg/24 h at last follow-up).

### 3.5. Non-Renal and Non-Bone Manifestations

Besides bone and renal disorder, 48% of cases presented symptoms involving different organs and apparatus: facial dysmorphisms (31%), intellectual disabilities/neurological abnormalities (7%), eyes/sight impairment (15%), in terms of corneal clouding, keratitis or exophthalmos, and hearing impairment (3%), in terms of conductive hearing impairment. Furthermore, isolated extra renal and extra bone symptoms were reported in 12% of patients, whereby diaphragmatic hernia, asthma, alopecia, aortic aneurysm, mitral valve regurgitation, furthermore in one case hypothyroidism and arachnodactyly were reported.

## 4. Discussion

MCTO is an ultrarare genetic condition characterized by progressive osteolysis in multiple skeletal sites often associated with nephropathy in more than half of the cases. In 2012, Zankl et al. [1] for the first time identified missense mutations clustering within a 51 base pair region of the single exon of MAFB in five unrelated simplex cases of MCTO. After that, 57 cases with genetic diagnosis were reported in the literature [1,2,3,4,5,6,7,8,9,10,11,12,13,14,15,16,17]. All cases showed a large phenotypical heterogeneity, even among patients presenting the same variant [4]. Furthermore, Dworschak et al. [7] suggested an incomplete penetrance of the disease, reporting three healthy carriers in the family of an affected individual. These results suggest a possible role for yet unidentified modifier gene(s), epigenetic mechanisms, or environmental factors.

Limited to nephropathy, our experience suggests that kidney involvement in MCTO is more frequent than expected (70% of cases), and similarly to bone disorder, kidney disease may present with a wide variety of clinical presentations, ranging from early development of kidney failure within the first decade of life to asymptomatic urinary abnormalities with prolonged preserved kidney function.

In this study we extended the spectrum of MCTO-associated nephropathy. In contrast to previous reports, we observed that nephropathy may onset within the first year of life, and furthermore, may present with proteinuria in nephrotic range. Extra-bone and extra-renal symptoms, considered so far exceptional, were reported by almost half of the subjects.

Nephrotic-range proteinuria was never accompanied by nephrotic syndrome, neither in the nine cases of FSGS [2,9,12,14]. The kidney involvement in MCTO may be explained by the essential role of *MAFB* gene in the function and differentiation of glomerular podocytes. More in detail, MafB is a basic leucine zipper transcription factor, expressed in both developing and mature podocytes. It regulates the later steps of podocyte development. In vivo studies on Mafb-deficient animal models demonstrated that MafB is essential for podocyte differentiation and foot processes formation [18]. Moreover, *MAFB* expression in podocytes is decreased in primary FSGS patients [19]. An enforced overexpression of MafB in podocytes limited the progression to chronic kidney damage in murine model of FSGS [19]. Overall, such findings suggested that MafB may have protective role against podocyte injury in CKD.

*MAFB* mutations causing MCTO disease were reported exclusively clustered in a specific domain of the gene. *MAFB* consists of two parts: the N-terminal transactivation domain, which is involved in MCTO disorder, and the C-terminal DNA-binding domain, including the leucine zipper required for dimerization and responsible of Duane retraction syndrome (DURS3; OMIM#617041) consisting in congenital ophthalmologic complications for the restriction of horizontal eye movements and eyeball retraction upon adduction with or without inner-ear defects [20]. Of note, a *MAFB* DNA-binding domain missense variant has been reported causing nephropathy also in association with DURS3, the FSGS-DURS3 disorder [21]. 

We report a 9-year-old male presenting with proteinuria in sub-nephrotic range and normal kidney function, with FSGS proven by kidney biopsy. As expected, nephrotic syndrome was steroid-resistant with limited response to ACE inhibitor. After administration of CyA (serum range lever 80–120 ng/dL) we reported a complete remission at 1 year of follow-up. So far, one patient with clinical diagnosis of MCTO was reported having partial remission of proteinuria after CyA administration at 1 year of follow-up [22]. Similar results were reported for the subject with FSGS-DURS3 treated with CyA [23]. To the best of our knowledge, we report for the first time a case of MCTO with complete remission of proteinuria after administration of CyA.

Although genetic forms of nephrotic syndrome are usually unresponsive to immunosuppressive drugs, proteinuria reduction has been reported after calcineurin inhibitors administration in genetic FSGS [24,25].

Although in most of genetic nephropathy the only therapeutic option is non-specific pharmacological proteinuria lowering with blockers of the renin-angiotensin-aldosterone system [26] or, more recently, with sodium-glucose cotransporter 2 inhibitors (SGLT2i) [27]. The association of CyA to the traditional anti-proteinuric therapy may represent an alternative option for patients with *MAFB*-associated nephropathy. 

According to the KDIGO guidelines [28] and the International Society of Pediatric Nephrology (IPNA) recommendations [29], immunosuppressive treatments are discouraged in monogenic forms of nephrotic syndrome/FSGS. However, the antiproteinuric effect of CyA was reported resulting, at least in part, from the maintenance of the integrity of the glomerular filtration barrier through its direct effect on actin-binding proteins in podocytes, as synaptopodin, essential in stabilizing the cytoskeleton and foot processes [30,31,32,33,34], that are altered MCTO. Similar effects may be speculated also for the FSGS-DURS3. Further mechanisms by which CyA acts on proteinuria are not clearly defined, although renal vasoconstriction (renal hemodynamic changes) and direct action on the permselectivity of the glomerular basement membranes, particularly on the electrochemical barrier that repels anionic albumin molecules may be suggested [35]. On the other hand, long-term common complications of CyA therapy, such as severe renal tubule-interstitial fibrosis are largely reported and may limit the drug’s administration [36]. Therefore, further studies to better define the role of CyA in such forms are needed. At Istituto Giannina Gaslini, an internal therapeutical protocol on the administration of CyA in MCTO was recently approved. In particular, the protocol allows the administration of CyA in all cases presenting with significative proteinuria (>1 g/d), after ineffective RAASi. Kidney biopsy at the beginning and after 3 years of CyA treatment is requested. 

As a major limitation we reported that retrospective nature of study may have limited the data collection, as example data on proteinuria were not available as urine protein/creatinine ratio.

As conclusion, MCTO should be considered early in patients with carpal and tarsal osteolysis to avoid misdiagnosis of arthritic disease and potential harmful treatment in patients at high risk of nephropathy. MCTO is characterized by a wide clinical variability of the kidney disease, that may appear early in life, manifesting with proteinuria even in nephrotic-range, with severe long-term outcomes and poor kidney survival in adulthood. 

The identification of the underlying gene mutation responsible for the disorder is crucial for the clinical management, appropriate renal follow-up and therapeutic approach. 

## Figures and Tables

**Table 1:** Patients’ characteristics of bone and kidney disease.

	Total (*n* = 54)	Literature pts (*n* = 42)	Survey pts (*n* = 12)
**Bone Disease**			
**Bone involvement, *n* (%)**	53 (98)	41 (98)	12 (100)
Age at first bone disease manifestation, years (IQR)	2 (1–4)	2 (1.5–4)	2.3 (0.9–6.25)
Misdiagnosis of reumathological disorder, *n* (%)	16 (30)	13 (32)	3 (25)
Age at MCTO diagnosis, years (IQR)	9.7 (5.1–15.7)	10.7 (5.1–14.7)	9 (6–16.25)
Median time from 1st bone manifestation to diagnosis, years (IQR)	6.2 (2.8–11.2)	7.2 (3.8–12.5)	6 (2–9)
**Treatment bone disease**			
Steroids, *n* (%)	4 (7.5)	3 (7.3)	1 (8.3)
NSAIDs, *n* (%)	8 (15)	7 (17)	1 (8.3)
DMARDs, *n* (%)	11 (21)	7 (17)	4 (33.3)
Denosumab, *n* (%)	7 (13.2)	2 (4.8)	5 (41.6)
**Kidney disease**			
**Kidney involvement**	38 (70.3)	28 (66.6)	10 (83.3)
Age at first kidney disease manifestation, years (IQR)	7 (4–13.7)	11 (4.5–15.5)	5 (3.2–9)
Median time from 1st bone manifestation, years (IQR)	5 (1–11.3)	9.5 (3.1–12.9)	2.1 (1–3)
Proteinuria	30 (79)	20 (100)	10 (100)
Nephrotic range proteinuria, *n* (%)	4 (13)	0 (0)	4 (40)
Non-nephrotic range proteinuria, *n* (%)	20 (67)	14 (70)	6(60)
Not available, *n* (%)	6 (20)	6 (30)	0 (0)
Microhematuria, *n* (%)	1 (3)	1 (4)	0 (0)
Chronic kidney disease at onset (eGFR < 90 mL/min/1.73 m^2^)	1 (3)	0 (0)	1 (10)
End-stage kidney disease at onset, *n* (%)	5 (13)	5 (18)	0 (0)
CAKUT, *n* (%)	2 (5.2)	1 (4)	1 (10)
**Renal Histology**			
Kidney Biopsy, *n* (%)	11 (28)	6 (28)	5 (50)
FSGS, *n* (%)	9 (81.8)	100 (6/6)	3 (60)
Mesangial abnormalities, *n* (%)	2 (18)	-	2 (40)
Tubulointerstitial abnormalities, *n* (%)	3 (27)	-	3 (60)
**Treatment kidney disease**			
RAASi, *n* (%)	12 (31.5)	5 (17.8)	7 (70)
Oral steroids, *n* (%)	3 (7.8)	1 (3.5)	2 (20)
Cyclosporine, *n* (%)	1 (2.6)	0 (0)	1 (10)
**Renal outcomes**			
Median follow-up time from renal disease onset, years (IQR)	9 (3–13)	7 (3–15)	11 (6–13)
End-stage kidney disease, *n* (%)	17 (44.7)	14 (50)	3 (30)
Median age at ESKD, years (IQR)	17 (9.3–20)	17 (10.5–20)	11 (11–22.5)
Median time from 1st renal manifestation to ESKD, years (IQR)	8.7 (4.5–12)	5.5 (3.3–9.2)	8.1 (8.1–12)
**Other Manifestations**			
Any extra renal/bone symptoms, *n* (%)	26 (48)	19 (45)	7 (58)
Facial dysmorphisms, *n* (%)	17 (65.4)	14 (73.6)	3 (42.8)
Intellectual disabilities/neurological abnormalities, *n* (%)	4 (15.4)	2 (10.5)	2 (28.5)
Eyes/sight impairment, *n* (%)	8 (30.7)	6 (31.5)	2 (28.5)
Hearing impairment, *n* (%)	2 (7.7)	2 (10.5)	0 (0)
Other symptoms, *n* (%)	7(26.9)	5 (26.3)	2 (28.5)

CAKUT, congenital anomalies of the kidney and urinary tract; DMARDs, disease modifying antirheumatic drugs; ESKD, end stage kidney disease; FSGS, focal segmental glomerulosclerosis; NSAIDs, non-steroidal anti-inflammatory drugs; RAASi, renin-angiotensin-aldosterone system inhibitors. Numbers represent absolute values (%) and median (interquartile range) as appropriate.

## Supplementary Materials

The following supporting information can be downloaded at: https://www.mdpi.com/article/10.3390/jcm11154423/s1, Table S1: Survey questionnaire. Figure S1: Disease-causing variants in MAFB gene. Reference [37] are cited in the Supplementary Materials.
