# Supplementary material for: Multicentric Carpotarsal Osteolysis Syndrome Associated Nephropathy: Novel Variants of MAFB Gene and Literature Review
Source: J Clin Med. 2022 Jul 29;11(15):4423. doi: 10.3390/jcm11154423 (PMC9369440; doi:10.3390/jcm11154423)
Supplement: Supplementary file 1 [file jcm-11-04423-s001.zip › jcm-1819398-supplementary.pdf]

Supplementary Material S1. Questionnaire of the survey on Multicentric CarpoTarsal Osteolysis syndrome (MCTO)

* Mandatory answers

1) SECTION 1

-Please enter your name* (blank space)
-Please enter your email address*(blank space)
-Please enter the name of your Hospital/Istitution, City, and Country*(blank space)

2) SECTION 2- Patient information

-Gender (multiple choice) M/F/Unknown
-Ethnicity (blank space)

3) SECTION 3- Genotype

-Did the patient receive a genetic testing? (multiple choice)
Yes, genetic analysis confirmed the diagnosis of MCTO
No, the patient received a clinical diagnosis of MCTO
Unknown
-Age at diagnosis (decimal years) (blank space)
-Mutation detected (blank space)
-Were parents tested for MAFB mutation? (multiple choice)
Yes, both parents resulted negative (sporadic case)
Yes, one parent resulted carrier (no clinical ̀symptoms of disease)
One parent is affected
No, parents were not tested
Unknown

4) SECTION 4- Phenotype (onset)

-Which was the first manifestation of MCTO disease? (multiple choice)
Bone disorder
Kidney disorder
Concomitant bone and kidney disorder
Other
-If other, please specify (blank space)

5) SECTION 5-Renal disease

-Age at first kidney symptoms (decimal years) (blank space)
-Severity of proteinuria at onset(multiple choice)
Absence of proteinuria
Sub-nephrotic range proteinuria
Nephrotic range proteinuria
Unknown/Not available
-Kidney function at onset (multiple choice)
GFR > 90 ml/min/1.73m2
60< GFR < 90 ml/min/1.73m2
30 < GFR < 60 ml/min/1.73m2
15< GFR < 30 ml/min/1.73m2
GFR < 15 ml/min/1.73m2 – ESRD
Unknown/Not available
-Hematuria (multiple choice) Yes no Unknown/Not available
-If a kidney biopsy was performed, please enter a short description of histopathological findings (blank space)
-Did the patient receive any treatment for the kidney disease? (multiple choice for each row)
Choices: Yes, with complete proteinuria remission/Yes, with proteinuria partial remission/yYes, with no response/No/Unknown
Rows: Corticosteroids/Calcineurin Inhibitors/MMF or other immunosuppressants/RAASi (ACE/ARB)

6) SECTION 6 – Bone disease

-Age at first bone symptoms(decimal years) (blank space)
-Which joint(s) were affected? (multiple choice for each row)
Choices: Yes, severe involvement/Yes, mild involvement/No/Unknown
Rows: Wrist-Carpal bones/Elbows/Ankle-tarsal bones/Hand fingers/Toes/Others
-Please enter a short description of bone disorder (blank space)
-Did the patient receive a previous misdiagnosis?(multiple choice)
Yes, rheumatological disorder
Yes, metabolic bone disease secondary to the kidney failure
No
Other
-Did the patient receive any treatment for the bone disorder? (multiple choice for each row)
Choices: Yes, with improvement/Yes, with NO improvement/No/Unknown
Rows: Nonsteroidal anti-inflammatory drugs (NSAIDs)/ Disease-modifying antirheumatic drugs (DMARDs)/ Biologic agents (TNF) /Corticosteroids /Bisphosphonates /Denosumab/Correctional surgery
-Did the patient show other symptoms? (multiple choice for each row)
Choices: Yes, severe/Yes, mild/No/Unknown
Rows: Intellectual impairment-mental disability/Eyes-sight abnormalities/hearing abnormalities/Facial dysmorphisms/others
- If other, please specify (blank space)

7) SECTION 7- Kidney outcomes

-Did the patient reach ESRD? (multiple choice)
Yes
No
Unknown
-If yes, please enter the age at ESRD (decimal years) (blank space)
-Please enter RRT history (periods on HD, PD, transplant; any major complications) (blank space)
-Age at last evaluation (decimal years) (blank space)
-Severity of proteinuria at last evaluation (multiple choice)
Absence of proteinuria
Sub-nephrotic range proteinuria
Nephrotic range proteinuria
Unknown/Not available
-Kidney function at last evaluation (multiple choice)
GFR > 90 ml/min/1.73m2
60< GFR < 90 ml/min/1.73m2
30 < GFR < 60 ml/min/1.73m2
15< GFR < 30 ml/min/1.73m2
GFR < 15 ml/min/1.73m2 – ESRD
Unknown/Not available
-Is the patient still alive? (multiple choice)
Yes
No
Unknown

Supp. Table S1: List of variants reported by literature and variants collected by ERKNET survey.

	Nucleodite change (NM_005461.5)	Aminoacid change	ClinVar (accession n.)	Published cases (n.)	Survey cases (n.)	Cases with kidney involvement n. (%)	ACMG classification*	Ref
1	c.161C>T	p.Ser54Leu	VCV000030773	3		1(33%)	P/LP	(9, 17)
2	c.161C>G	p.Ser54Trp		1	1	1 (50%)	LP	(4)
3	c.167C>T	p.Ser56Phe		1			P	(7)
4	c.172A>G	p.Thr58Ala			1	1 (100%)	LP	present paper
5	c.173C>G	p.Thr58Arg	VCV000684763	1	1	2 (100%)	P	(15)
6	c.176C>T	p.Pro59Leu		5		4 (80%)	P	(1 ,4, 5, 8)
7	c.183C>A	p.Ser61Arg		1		1 (100%)	LP	(12)
8	c.184A>G	p.Thr62Ala	VCV000807627		1	1 (100%)	P	present paper
9	c.185C>T	p.Thr62Ile	VCV000803608	1	1	2 (100%)	P	(5)
10	c.188C>T	p.Pro63Leu		4	4	6 (75%)	P	(4, 9)
11	c.188C>G	p.Pro63Arg		3		3 (100%)	P	(2, 4)
12	c.194G>T	p.Ser65Ile		1		1 (100%)	LP	(4)
13	c.197C>G	p.Ser66Cys		1		1 (100%)	P	(4)
14	c.206C>T	p.Ser69Leu	VCV000030769	9	1	5 (50%)	LP	(3, 16, 5)
15	c.208T>C	p.Ser70Pro			1		P	present paper
16	c.209C>T	p.Ser70Leu	VCV000030770	1		1 (100%)	P	(5)
17	c.211C>G	p.Pro71Ala		2		2 (100%)	P	(12)
18	c.211C>T	p.Pro71Ser	VCV000030771	3	1	2 (50%)	P	(1, 5)
	Variant not reported			5		4 (80%)		(11, 13, 14)

(*) ACGM Classification: classification of the variant according to the American College of Medica Genetics and Genomics guideline.
(Genetics in Medicine, 2015; 17 pp.405-424). LP: Likely Pathogenic; P: Pathogenic.

Supp.Mat. Figure S1

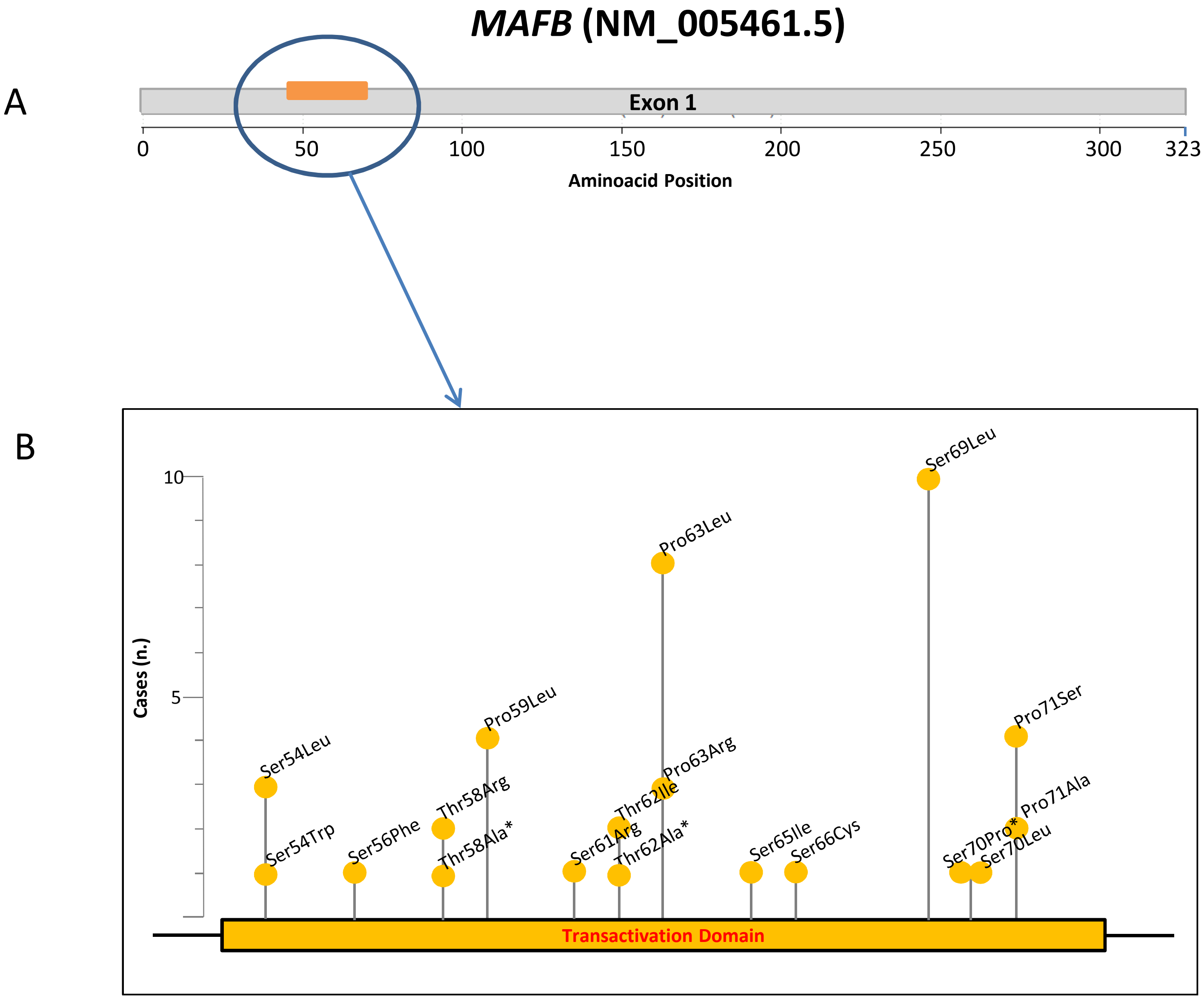

Legend: (A) schematic representation of the *MAFB* gene; in orange the transactivation domain contains cluster of missense variants. (B) Distribution of identified variant and number of observed cases. (*) New variants described in the present manuscript
